# Dietary Supplementation of *Lactobacillus reuteri* Modulates Amino Acid Metabolism and Extracellular Matrix in the Gut–Liver Axis of Weaned Piglets

**DOI:** 10.3390/ani15111567

**Published:** 2025-05-27

**Authors:** Yiyi He, Yangyang Wei, Shihui Ruan, Qiwen Wu, Yunxia Xiong, Li Wang, Zongyong Jiang, E Xu, Hongbo Yi

**Affiliations:** 1 Key Laboratory of Animal Genetics, Breeding and Reproduction in the Plateau Mountainous Region, Ministry of Education, College of Animal Science, Guizhou University, Guiyang 550025, China; yiyihe226@163.com; 2 State Key Laboratory of Swine and Poultry Breeding Industry, Key Laboratory of Animal Nutrition and Feed Science in South China, Guangdong Key Laboratory of Animal Breeding and Nutrition, Ministry of Agriculture and Rural Affairs, Institute of Animal Science, Guangdong Academy of Agricultural Sciences, 1 Dafeng 1st Street, Guangzhou 510640, China; 15878672059@163.com (Y.W.); jodieyuen@163.com (S.R.); wuqiwen@gdaas.cn (Q.W.); xiangtang.2000@163.com (Y.X.); wangli1@gdaas.cn (L.W.); jiangzy@gdaas.cn (Z.J.)

**Keywords:** amino acid metabolism, extracellular matrix, weaned piglets, *Lactobacillus reuteri*

## Abstract

Weaning stress leads to intestinal dysfunction and impaired growth performance and intestinal development in piglets. This study aims to investigate the effects of *Lactobacillus reuteri* LR1 on growth performance and amino acid metabolism in the gut–liver axis of weaned piglets. A total of 48 weaned piglets (*Duroc × Landrace × Yorkshire*, 21 days old) were randomly assigned to the CON group (fed a basal diet) and the LR1 group (fed the basal diet supplemented with 5 × 10^10^ CFU/kg of *Lactobacillus reuteri* LR1) with six pens per group and 4 piglets each pen. The results demonstrated that LR1 significantly increased average daily gain (ADG), average daily feed intake (ADFI), and final body weight (*p* < 0.05). Additionally, LR1 significantly enhanced the villus height of the ileum (*p* < 0.05) and upregulated the expression of SLC6A19 in the jejunum, as well as SLC6A19, SLC7A1, and SLC38A9 in the ileum (*p* < 0.05). Amino acid analysis revealed that LR1 elevated the serum concentrations of glycine and hydroxyproline, along with increased taurine in the liver. Masson staining indicated LR1 reduced ileum fiber deposition, with COL3A1 identified as a key component. Furthermore, untargeted metabolomic analysis identified 27 amino acid-related differential metabolites and 11 significantly up-regulated in the plasma of the hepatic portal vein, including L-asparagine, L-citrulline, His-Cys, N-acetyltryptophan, 4-hydroxy-l-isoleucine, Gly-Arg, creatine, ornithine, ectoine, 3-methyl-l-histidine, and stachydrine. Correlation analysis suggested that COL1A2 and COL3A1 were closely associated with these metabolic changes. Overall, these findings suggest that LR1 supplementation promotes growth, improves intestinal morphology, reduces fiber deposition, and enhances amino acid metabolism in the gut–liver axis of weaned piglets.

## Simple Summary

This study demonstrates that dietary supplementation with *Lactobacillus reuteri* LR1 alleviates weaning stress in piglets by enhancing growth and gut–liver axis metabolism. In a 21-day trial with 48 weaned piglets, LR1 significantly improved growth performance, and LR1 enhanced intestinal nutrient absorption via increased ileum villus height and upregulated amino acid transporters. Metabolically, LR1 elevated serum glycine, hydroxyproline, and hepatic taurine levels, with untargeted metabolomics identifying 11 amino-acid-related metabolites upregulated in portal plasma. Histological analysis revealed reduced ileum collagen deposition, and correlation analysis linked collagen dynamics to gut–liver axis amino acid metabolic reprogramming. This study highlights LR1 as a promising probiotic for optimizing nutrient utilization and promoting intestinal development in piglets, and the intestinal extracellular matrix may be a key target for improving amino acid transport in the gut–liver axis of weaned piglets.

## 1. Introduction

In modern swine production, the healthy development of weaned piglets is fundamental to economic profitability in the pig farming industry. However, piglets encounter numerous challenges during their growth [1]. Weaning stress in piglets leads to growth retardation, digestive disorders, and immune suppression, significantly affecting production efficiency and mortality rate [2,3,4]. Therefore, promoting the growth of piglets and improving production performance are not only conducive to promoting the animal welfare of piglets but also to reducing economic loss on farms.

The gut–liver axis represents a bidirectional communication system between the gastrointestinal tract and the liver, influencing physiological and pathological processes in animals significantly [5]. The foundation of piglet growth and development depends on the organism’s ability to efficiently absorb and metabolize amino acids [6,7]. The liver plays a central role in regulating amino acid distribution, as it receives the majority of amino acids transported via the portal vein [8]. Thus, the efficiency of amino acid transport within the gut–liver axis is a key determinant of overall amino acid absorption. However, intestinal barrier dysfunction, such as increased intestinal permeability, allows microbial endotoxins and metabolites to translocate into the portal circulation, triggering hepatic inflammation and oxidative stress [9,10]. Previous studies have found that weaned piglets show high diarrhea and mortality rates due to the underdeveloped digestive and absorptive functions of their intestines [11,12]. Therefore, proper intestinal development is particularly crucial for improving the growth performance and survival of piglets. Probiotics and phytogenic compounds have demonstrated therapeutic potential by suppressing harmful bacterial populations, promoting SCFA synthesis, and inhibiting hepatic stellate cell activation [13,14]. In addition, a previous study demonstrated that *Lactobacillus reuteri* LR1, which was renamed *Limosilactobacillus reuteri* (*L. reuteri*) in 2020 [15], significantly increases amino acid transport in the ileum of weaned piglets, and this process was highly correlated with changes in the extracellular matrix (ECM) [16]. The ECM provides essential structural support for intestinal cells, regulates cellular behavior, participates in signaling pathways, and contributes to the maintenance of the intestinal barrier [17]. The density and stiffness of the ECM are strongly associated with amino acid uptake and protein synthesis in surrounding cells [18]. ECM stiffening activates glycolysis and glutamine metabolism surrounding fibroblasts mechanically and thus coordinates amino acid flux within the microenvironment [19,20]. Therefore, this experiment was conducted to investigate the effects of LR1 on intestinal amino acid metabolism and the intestinal health of weaned pigs, with a view to providing a theoretical basis for the nutrition of piglets.

## 2. Materials and Methods

All animal protocols applied in this study complied with the Guidelines for the Care and Use of Animals for Research and Teaching at Guangdong Academy of Agricultural Sciences. They were also approved by the Animal Care and Use Committee of the Guangdong Academy of Agricultural Sciences.

### 2.1. Animals and Samples

In this experiment, 48 weaned piglets (*Duroc* × *Landrace* × *Yorkshire*), 21 days old with an average body weight of 5.1 kg, were randomly assigned to two groups with balanced sex distribution. Each group had 6 pens, with 4 piglets in each pen. The control group (CON) was fed a basal diet. The *Lactobacillus reuteri* group (LR1) received the basal diet supplemented with 5 × 10^10^ CFU/kg *Lactobacillus reuteri* [21]. The basal diet was formulated as a powdered compound feed based on the nutrient requirements of swine outlined by the NRC (2012), and its composition and nutritional levels are shown in Table 1. The probiotic freeze-dried powder was diluted and thoroughly mixed with feed. The experimental period lasted 21 days. Piglets were fed four times at 08:00, 12:00, 16:00, and 20:00 every day, and the diarrhea situation of piglets was observed and recorded. After the experimental period, 1 piglet was randomly selected from each pen and euthanized. Samples of small intestines, livers, and hepatic portal vein plasma were collected. Samples for section preparation were stored in 4% paraformaldehyde, while the remaining samples were stored in a −80 °C freezer.

### 2.2. Quantitative Real-Time PCR

The total RNA of the jejunum and ileum was extracted using TRIzol reagent, and the total RNA concentration was determined. Then, the cDNA was synthesized following the instructions provided in the reverse transcription kit. Primers specific to the target genes were designed based on gene sequences using the Primer 5.0 software and subsequently synthesized after verification through the NCBI database. The corresponding primer sequences are shown in Table 2. cDNA was prepared in a real-time fluorescence quantitative PCR reaction system, and the relative expression of the mRNA of the target genes was calculated by the 2^−ΔΔCt^ method according to the threshold cycling of the internal reference genes and the target genes’ expression; then, the expressions for each target gene were normalized to the control piglets (1.0) [22].

### 2.3. H&E Staining & Masson Staining

Small intestinal tissues fixed in 4% paraformaldehyde were paraffin-embedded, followed by 4 μm sections and hematoxylin–eosin (H&E) staining. The stained sections were placed under an Axio Scope A1 microscope (Zeiss, Germany) and photographed; the height of the villi and the depths of crypts were counted by using the Image J software win64; and the ratio of the height of the villi to the depth of the crypts was calculated [23].

After staining the nuclei of the above sections with hematoxylin staining solution, the sections were differentiated with hydrochloric acid alcohol and then rinsed with ultrapure water. The sections were immersed in Masson Ponceau acid fuchsin solution and later rinsed with ultrapure water, washed in aqueous glacial acetic acid, and then dehydrated with an alcohol gradient, followed by immersion in xylene solution for clearing. Finally, the sections were sealed with neutral gum and observed using an Axio Scope A1 microscope, and the collagen volume fraction was calculated using Slide Viewer 2.5 and Image J [24].

### 2.4. Free Amino Acids Concentration Analysis

Fresh liver tissue was collected from piglets. The tissue was mixed with hydrochloric acid, ground by a grinder, and then centrifuged at 4 °C at 12,000 rpm for 15 min. The supernatant was collected, and 10% sodium sulfosalicylate was added to the supernatant (3:1, *v*/*v*). The mixture was thoroughly mixed and subjected to a second round of centrifugation. The supernatant was then collected using a sterile syringe, filtered, and transferred into an injection vial for further analysis. Plasma samples were directly mixed with 10% sodium sulfosalicylate, followed by centrifugation to obtain the supernatant. The supernatant was subsequently filtered and transferred into a sample vial for testing. The amino acid concentration of the samples was measured using a fully automated amino acid analyzer (L-8900, Hitachi, Ltd., Tokyo, Japan), and the results were processed and analyzed using its accompanying the EZChrom Elite software (version 3.1.5.b); then, the final results were expressed as nmol/μL. Buffer preparation referenced Yang [25].

### 2.5. Untargeted Metabolomics Analysis

Blood samples were collected in Vacutainer tubes containing EDTA and centrifuged at 1500× *g* for 15 min at 4 °C. The plasma was then aliquoted into 150 μL portions and stored at −80 °C until liquid chromatography–mass spectrometry (LC-MS) analysis. Prior to analysis, plasma samples were thawed at 4 °C. A 100 μL aliquot was mixed with 400 μL of cold methanol–acetonitrile (1:1, *v*/*v*) to precipitate proteins. The mixture was centrifuged at 14,000× *g* for 20 min at 4 °C, and the resulting supernatant was dried using a vacuum centrifuge. The dried extract was then reconstituted in 100 μL of acetonitrile–water (1:1, *v*/*v*) and centrifuged at 14,000× *g* for 15 min at 4 °C, and the supernatant was injected for analysis.

Analysis was performed using a UHPLC (Vanquish UHPLC, Thermo, Waltham, MA, USA) coupled to an Orbitrap (Shanghai Applied Protein Technology Co., Ltd., Shanghai, China). The raw MS data were converted into MzXML files using ProteoWizard MSConvert 3.0.22170 before importing into freely available XCMS software 3.22.0. Compound identification of metabolites was performed by comparing the accuracy (*m*/*z*) value (<10 ppm) and MS/MS spectra with an in-house database established with available authentic standards.

### 2.6. Statistical Analysis

Statistical analysis was performed using the SPSS 26.0 software, and the data were expressed as “mean ± SEM”. Statistical treatment was performed using Student’s *t*-test, with *p* < 0.05 considered statistically significant. VIP > 1 and a *p*-value < 0.05 were used to screen significantly changed metabolites; Pearson’s correlation analysis was performed to determine the correlation between two variables in untargeted metabolomics analysis.

## 3. Results

### 3.1. Growth Performance and Intestinal Morphology of Weaned Piglets

As shown in Table 3, LR1 significantly increased the final body weight, average daily gain (ADG), and average daily feed intake (ADFI) of piglets compared to the CON group (*p* < 0.05). However, no significant differences were observed in the ADG-to-ADFI ratio and the incidence of diarrhea between the LR1 group and the CON group.

**Figure 1:**
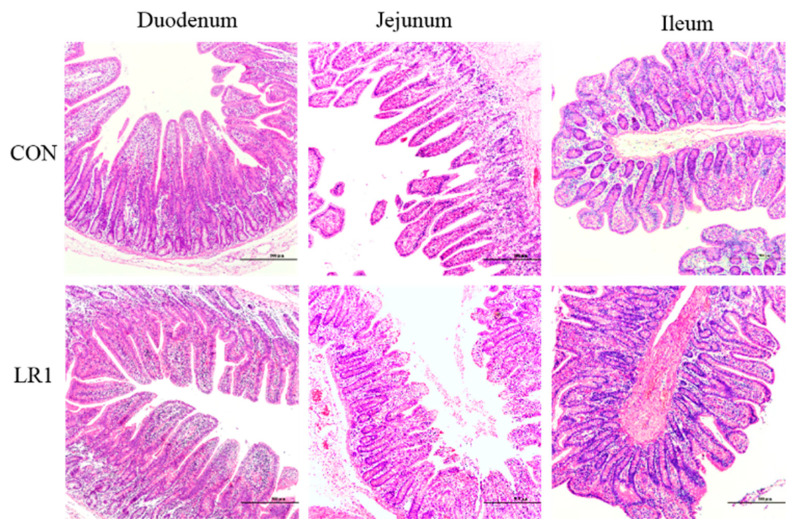
H&E-stained sections of duodenum, jejunum, and ileum of the piglets. The magnification of the picture is 10 times, and the scale is 500 μm.

The H&E-staining sections of the duodenum, jejunum, and ileum of the piglets are shown in Figure 1. Compared with the CON group, in the LR1 group, the height of the intestinal villus was significantly increased in the ileum (*p* < 0.05) but exhibited a significantly lower villus-to-crypt ratio in the duodenum (*p* < 0.05), as shown in Table 4.

### 3.2. Amino Acid Transport in the Gut–Liver Axis of Piglets

**Figure 2:**
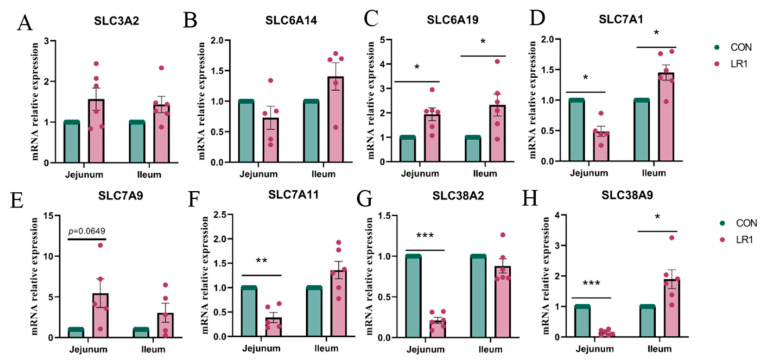
mRNA expression of amino acid transport carriers in the intestine of piglets. The mRNA expression of SLC3A2 (**A**), SLC6A14 (**B**), SLC6A19 (**C**), SLC7A1 (**D**), SLC7A9 (**E**), SLC38A2 (**F**), SLC38A2 (**G**), and SLC38A9 (**H**) in the jejunum and ileum. The values are presented as mean ± SEM, n = 6. * *p* < 0.05, ** *p* < 0.01, and *** *p* < 0.001.

To evaluate the effect of LR1 on amino acid transport in the gut–liver axis of weaned piglets, the expression of amino acid transport carrier mRNA in the jejunum and ileum of piglets was investigated using q-PCR. The results, presented in Figure 2, indicate that LR1 supplementation significantly increased the mRNA expression of SLC6A19 (Figure 2C) while significantly decreasing the expression of SLC7A1 (Figure 2D) and markedly downregulating SLC7A11, SLC38A2, and SLC38A9 (Figure 2F–H) in the jejunum compared to the CON group. In addition, in the ileum, LR1 supplementation significantly upregulated the expression of SLC6A19, SLC7A1, and SLC38A9 (Figure 2C,D,H) compared to the CON group. However, no significant changes were observed in the expression of SLC3A2 and SLC6A14 in the intestine.

Subsequently, the free amino acid concentrations in the hepatic portal vein sera and livers of weaned piglets were measured by automatic amino acid analyzers. Table 5 shows the free amino acid concentrations in the sera of the experimental piglets. The results show that LR1 significantly increased the concentrations of glycine and hydroxyproline in the portal vein serum and decreased the concentrations of alanine, cysteine, glutamine, histidine, and isoleucine. In contrast to the changes in the portal vein serum, the amino acid concentrations in the liver showed different patterns. In the liver (Table 6), LR1 increased the level of taurine and reduced the concentrations of isoleucine, alanine, leucine, lysine, aspartic acid, ammonia, ornithine, phenylalanine, proline, cysteine, serine, taurine, threonine, glutamic acid, glutamine, histidine, and valine significantly.

### 3.3. Structural Changes in the Extracellular Matrix of the Piglet Intestine

**Figure 3:**
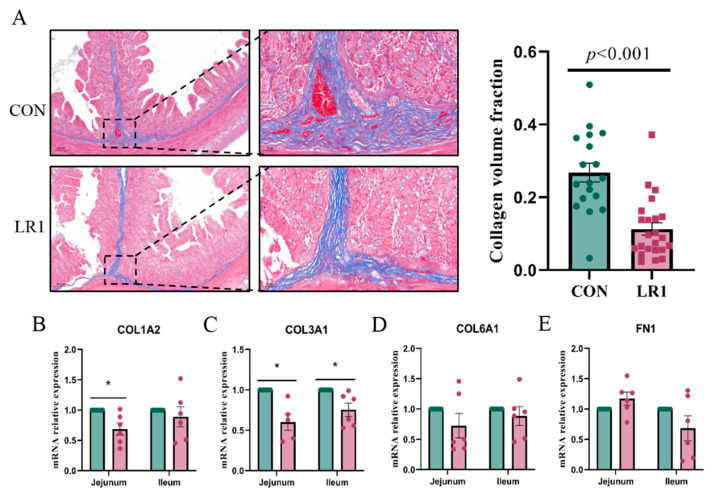
Effects of LR1 on the structure of the extracellular matrix in the intestine. (**A**) Masson-stained sections of the ileum of weaned piglets (left) and the collagen volume fraction of the ileum. mRNA expression of COL1A2 (**B**), COL3A1 (**C**), COL6A1 (**D**), and FN1 (**E**) in the jejunum and ileum. The values are presented as mean ± SEM, n = 6. * *p* < 0.05.

To investigate the effect of LR1 on the extracellular matrix of the intestinal mucosa of weaned pigs, Masson staining was performed to assess the collagen fiber content in the ileum. The sections were then quantified using the Image J software (Figure 3A, left), and the ratio of collagen fibers (blue) to the total tissue surface area was calculated to determine the collagen volume fraction in the ileum. The result showed that CVF was reduced significantly in the LR1 group (Figure 3A, right). Furthermore, the mRNA expression levels of key molecules in the extracellular matrix of the jejunum and ileum were analyzed. The results showed that LR1 significantly decreased the expression of COL1A2 (Figure 3B) and COL1A3 (Figure 3C) in the jejunum, but it had no significant impact on the expression of COL6A1 (Figure 3D) or FN1 (Figure 3E). In the ileum, LR1 significantly reduced the expression of COL1A3 but had no significant impact on the expression of COL1A2, COL6A1, or FN1. The results indicated that LR1 significantly decreased the volume fraction of collagen in the ileum of piglets, suggesting that LR1 can reduce the deposition of collagen fibers in the ileum of weaned piglets.

### 3.4. Metabolomics Analysis of Hepatic Portal Vein Plasma in Piglets

**Figure 4:**
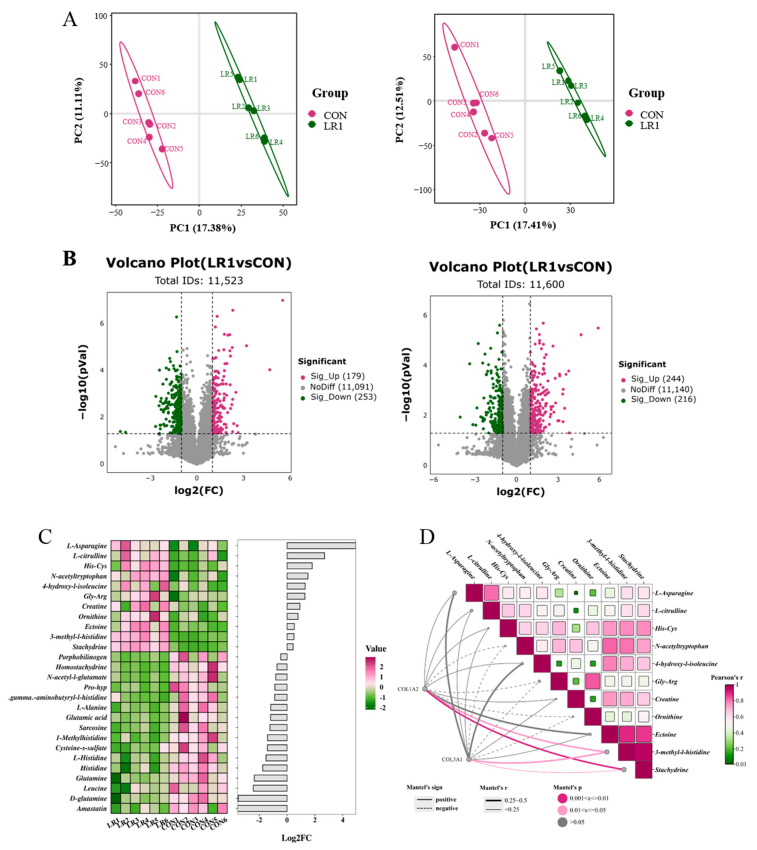
Effects of LR1 on the metabolites of hepatic portal vein plasma in weaned piglets. (**A**) Score plot of PLS-DA in positive (**left**) and negative (**right**) ion modes. (**B**) Volcano plot in positive (left) and negative (right) ion modes. (**C**) Heatmap and histogram of significantly differential metabolites; red color represents high expression, green color represents low expression, and the abscissa is the log2 (FC) of the metabolites in the histogram; VIP > 1, *p* < 0.05. (**D**) Correlation network heatmap analysis between the extracellular matrix and amino acid metabolites. Pearson correlation analysis was used for the heatmap, and the network diagram was analyzed using the Mantel test: linear representation of positive relationships between matrices (r sign); *p* < 0.05 was regarded as a significant correlation between the two matrices, represented by a solid line; *p* ≥ 0.05 indicated no significant correlation, represented by a dotted line. The larger the absolute value of r (r.abs), the stronger the correlation between the two matrices.

To investigate the impact of incorporating LR1 into the diet on nutrient metabolism in the digestive tract of underweight pigs, a non-targeted metabolomics analysis was used to assess changes in metabolites within the porcine hepatic portal vein plasma of the piglets. Figure 4A presents a partial least squares discriminant analysis (PLS-DA) plot for samples from both groups. The model evaluation parameters obtained through 7-fold cross-validation yielded a positive Q2 value of 0.617 (left) and a negative Q2 value of 0.543. Q2 > 0.5, indicating that this model is robust and that there are significant differences in metabolite compositions between samples from the two groups. Metabolites exhibiting a fold change (FC) greater than 1.5 or less than 0.67, along with a *p*-value below 0.05, were classified as differential metabolites and are visually represented using a volcano plot (Figure 4B). In total, 11,523 metabolites were detected in the positive ion mode, with 179 significantly upregulated and 253 significantly downregulated in the LR1. Furthermore, 11,600 metabolites were detected in the negative ion mode, with 244 significantly upregulated and 216 significantly downregulated in the LR1. After combining the amino acid metabolites detected under both modes, significantly differential metabolites were screened based on VIP > 1 and *p* < 0.05 (Figure 4C). The LR1 group showed a significant increase in the contents of 11 metabolites, including L-asparagine, L-citrulline, His-Cys, N-acetyltryptophan, 4-hydroxy-l-isoleucine, Gly-Arg, creatine, ornithine, ectoine, 3-methyl-l-histidine, and stachydrine.

In the above results, LR1 significantly increased the villus height of the ileum in weaned piglets and significantly reduced the CFV in the ileum. To explore the potential link between enhanced amino acid metabolism and these intestinal structural changes, a correlation network heatmap analysis (Figure 4D) was conducted using COL1A2 and COL3A1. The results showed that the decreases in COL1A2 and COL3A1 were closely related to the upregulation of these 11 metabolites. Notably, the downregulation of COL1A2 and COL3A1 exhibited a positive connection with the upregulation of eight metabolites, L-asparagine, L-citrulline, His-Cys, 4-hydroxy-l-isoleucine, creatine, ectoine, 3-methyl-l-histidine, and Stachydrine, and the down-expression of COL1A2 and COL3A1 showed significantly positive correlations with 3-methyl-l-histidine and stachydrine.

## 4. Discussion

Following the restriction on antibiotic use in animal feed, researchers have proposed various alternatives to fill the gap left by antibiotic growth promoters [26]. Studies have shown that intestinal probiotics are critical to host functions such as nutrient absorption and immunity [27,28]. Gut-derived metabolites, including SCFAs, enhance intestinal barrier function, reducing endotoxin translocation and oxidative stress in the liver [29]. Microbial imbalances elevate trimethylamine N-oxide and lipopolysaccharides, exacerbating hepatic steatosis and fibrosis conversely [30], even impairing neural function and behavioral adaptability [31]. Gut microbiota modulate cardiovascular health through their metabolites, which drive endothelial dysfunction and atherosclerosis [32]. Probiotics restore microbial balance, mitigating myocardial injury via anti-inflammatory pathways [33]. Gut microbes synergistically regulate the gut–liver, gut–brain, and gut–heart axis through metabolites and inflammatory signaling; systemic oxidative stress and probiotic interventions are key mediators of cross-organ health interactions. The above studies highlight that gut-microbiota-derived metabolites critically support organ function and systemic physiological integration.

LR1 has been recognized for its role in maintaining intestinal microbial balance and inhibiting the proliferation of pathogenic bacteria, thereby enhancing the growth performance of piglets, improving intestinal immunity, and promoting gut health [34]. Previous studies have demonstrated that LR1 supplementation produces effects comparable to antibiotic-containing feed in improving the growth performance of 21-day-old weaned piglets [22]. Another study found that LR1 can inhibit the apoptosis of intestinal epithelial cells and maintain the integrity of the intestinal wall in mice by regulating the expression of intestinal tight junction proteins [35]. This indicates that LR1 may potentially serve as one of the feed additives used to replace antibiotics. In addition, LR1 demonstrated the same promoting effect on the growth performance of piglets and had a dominant position within *Lactobacillus* [36,37]. In our study, the addition of LR1 to the diet significantly increased the final body weight, average daily feed intake, and average daily gain of piglets, remarkably improving the growth performance of piglets, which is also consistent with previous research findings. Although there were no significant effects on the G/F and diarrhea of piglets, there were positive trends in both aspects. Furthermore, LR1 significantly reduced the V/C of the duodenum and significantly increased the villus height of the ileum, which is similar to the research results of Tang [36]. This indicates that LR1 has the potential to be used as an alternative to antibiotic feed additives to promote the growth of piglets and improve intestinal health.

The liver is the governor of the flow of most amino acids in animals, and the total number of amino acids pooled in the hepatic portal vein determines the uptake of amino acids by the animal organism [8]. Yang’s [25] experiment found that LR1 could significantly increase the gene expression of SLC6A19 and SLC38A9 in the ileum, as well as the expression of SLC7A1 in the duodenum. The exogenous addition of supplements to the sow’s diet can likewise boost the expression of SLC7A1 in the piglets’ intestines [38]. In this study, LR1 significantly increased the expression of SLC6A19 in the jejunum and SLC6A19, SLC7A1, and SLC38A9 in the ileum, which is consistent with the abovementioned research results. This may imply that LR1 can regulate the physiological processes that facilitate the flow of amino acids from the intestine to the liver. The different expression trends of SLC7A1 in the jejunum and ileum and the decrease in the concentrations of SLC38A2 and SLC38A9 in the jejunum may be caused by the differences between different intestinal segments [39]. Collectively, these findings imply that LR1 has a positive effect on amino acid transport in the gut–liver axis.

The serum amino acids of piglets play a crucial role in growth performance, as well as in the development of gut health and important biological processes [40]. Consequently, a further assessment of the impacts of LR1 supplementation on the composition of serum amino acids was carried out. The results indicated that LR1 predominantly leads to a reduction in the levels of alanine, cysteine, glutamine, histidine, and isoleucine and increased glycine and hydroxyproline in the piglets’ serum. These findings concurred with a prior study demonstrating that the oral administration of Lactobacillus increases the glycine concentration in the sera of piglets [41]. Another study with similar results reported that the supplementation of nucleotides has a regulatory effect on the relative quantities of glutamic acid, aspartic acid, serine, alanine, glycine, leucine, and valine in the muscle of grass carp [42]. The observed decrease in certain free amino acid concentrations in both the serum and liver suggests that LR1 may contribute to the balance of the intestinal microecology and enhance nutrient absorption efficiency [43]. This improvement enables low-body-weight piglets to absorb amino acids more efficiently and rapidly. After absorption, amino acids are transported more quickly to other tissues and organs for metabolism and synthesis rather than accumulating in the serum of the hepatic portal vein and the liver [44]. Consequently, this process results in reduced amino acid levels in those parts [45]. In our research, the mRNA expression of amino acid transporters was upregulated by LR1, corroborating this hypothesis. On the other hand, it is possible that LR1 enhanced the deamination of amino acids, converting them into keto acids and ammonia [46]. Glutamic acid, serving as a specific precursor for the production of alanine, aspartic acid, proline, ornithine, arginine, and the antioxidant glutathione, can undergo transamination metabolism under the action of corresponding aminotransferases and dehydrogenases [47]. Ammonia is further synthesized into urea in the liver and excreted from the body, while keto acids enter other metabolic pathways to provide energy or synthesize other substances, thereby reducing the amino acid content in the liver [48]. This hypothesis is further supported by our observation of a tendency toward increased urea concentrations in the serum.

ECM is not only the supporting framework for various intestinal cells but also an important medium for the growth, differentiation, migration, and information transmission of these cells in the intestine [49]. When amino acids are restricted, ECM internalization caused by cells or degradation can upregulate the levels of intracellular tyrosine and phenylalanine, and the enhanced metabolism of tyrosine leads to an increase in fumarate, which promotes the TCA cycle and provides an energy source for the metabolic activities of cells [50,51]. LR1 decreases the CFV of the ileum, which may be caused by ECM internalization. Furthermore, collagen is the most abundant structure of the intestinal extracellular matrix, mainly distributed in the basement membrane [52]; the significant decrease in the collagen content demonstrates that LR1 promotes the internalization of ECM deeply in this study. To investigate the connection between changes in the ECM and amino acid metabolism, a correlation analysis was conducted. A positive association was observed between the downregulation of ECM components and the upregulation of amino acid metabolites, which might be attributed to the indirect promotion of amino acid transporter expression caused by the ECM through the activation of the YAP/TAZ pathway, as suggested in previous studies [53]. The results above indicate that the ECM could be an important target for improving intestinal amino acid metabolism in weaned piglets.

## 5. Conclusions

This study demonstrates that LR1 significantly improves growth performance in weaned piglets by upregulating intestinal amino acid transporter expression to enhance gut–liver axis amino acid transport while suppressing ECM component synthesis to alleviate ileum fibrosis. Correlation analysis identifies ECM remodeling as a pivotal regulatory target for the LR1-mediated modulation of amino acid metabolism. These findings establish a theoretical foundation for optimizing weaned piglet management and provide mechanistic insights for subsequent nutritional interventions.

## Figures and Tables

**Table 1:** Ingredients and composition of experimental diets (air-dry basis).

Ingredients	Proportion (%)	^2^ Nutrient Component	Content
Corn	58.06	NE(Kcal/kg)	2589
Soybean meal	16.07	CP	22.49
Low protein whey powder	8.00	SID Protein	18.94
Whey powder concentrate	10.00	CF	1.92
Fish meal	3.00	EE	3.56
Soybean oil	1.00	SID Lysine	1.46
Sucrose	1.00	SID Threonine	0.86
50% choline chloride	0.20	SID Methionine	0.47
NaCl	0.40	SID (Methionine + Cysteine)	0.81
L-lysine	0.22	SID Tyrosine	0.29
DL-methionine	0.08	SID Isoleucine	0.95
L-threonine	0.01	SID Valine	1.00
ZnO	0.20	SID Leucine	1.91
Limestone	0.44	SID Phenylalanine	0.83
Calcium hydrogen phosphate	0.30	Total Ca	0.49
Phytase	0.02	STTD Ca	0.33
^1^ Premix	1.00	Total P	0.41
Total	100	STTD P	0.26

^1^: Premix provided the following per kg of diets: VA, 9920 IU; VD3, 2240 IU; VK3, 4 mg; VB1, 2.4 mg; VB2, 8 mg; VB6, 6.4 mg; VB12, 32 mg; Nicotinic, 32 mg; pantothenic acid, 12 mg; folic acid, 0.8 mg; biotin, 64 mg; Fe 90 mg; Cu, 12 mg; Mn, 52.5 mg; I, 0.525 mg; Se, 0.36 mg. ^2^: Crude protein, total calcium, and total phosphorus are analyzed values; the rest are calculated values. NE = net energy; CP = crude protein; CF = crude fiber; EE = ether extract; SID = standardized ileal digestibility; STTD = standardized total tract digestible.

**Table 2:** Primer sequence information.

Genes	Primer Sequence (5′-3′)	Product Size/bp	Accession Numbers
*β-actin*	F: CTGCGGCATCCACGAAACT	380	XM_003124280
	R: AGGGCCGTGATCTCCTTCTG		
*COL1A2*	F: GTCTTGGCGGGAACTTTG	338	NM_001243655.1
	R: ACCGTTGTGACCCCTAATG		
*COL3A1*	F: TTTCAAAATCAACACCGACG	173	NM_001243297.1
	R: CTTGGTTAGGATCAACCCAATA		
*COL6A1*	F: ACTCAGAACAACCGGATCG	177	XM_021071094.1
	R: GCCTTGGCAGGAAATGAC		
*FN1*	F: AGCAAGAAGGACAATCGGGG	325	XM_003133642.5C
	R: AGTTGCCACCGTAAGTCTGG		
*SLC3A2*	F: AGCAGCGTGACTGTGAAGG	174	NM_001012662.2
	R: TGGCGGATGTAGGAGAAGAG		
*SLC6A14*	F: CCTTGGTCTCGTCTGTGTGA	136	NM_001348402.1
	R: TCTGTTCCCTCCATAAATCCA		
*SLC6A19*	F: TCATCTTCCTCTTCTTCTTCGTG	155	XM_003359855.4
	R: CTTGACCTTCTGGGATTTGG		
*SLC7A1*	F: TCTGGTCCTGGGCTTCATAA	192	NM_001012613
	R: ACCTTCGTGGCATTGTTCAG		
*SLC7A9*	F: GCCTATCAAGGTGCCCATC	144	NM_001110171.1
	R: AGCGGACGAACAGGAAGTAA		
*SLC7A11*	F: TGAATGGTGGTGTGTTTGCT	101	NM_014331.3
	R: AGTGTGTTTGCGGATGTGAA		
*SLC38A2*	F: TCCATTTGATGCCAGTGTTG	212	XM_005664159
	R: CTCAGAAGAACCAGCGAGGA		
*SLC38A9*	F: TCCTGCTGTTCCAGATGATG	180	NM_001349383.1
	R: TGATTCCTCCTATGTTTGGGTAG		

**Table 3:** Effects of LR1 on the growth performance of weaned piglets.

Item	CON	LR1	*p*-Value
Initial BW (kg)	5.11 ± 0.09	5.09 ± 0.08	0.887
Final BW (kg)	10.91 ± 0.22 ^b^	11.86 ± 0.32 ^a^	0.036
ADFI (g/d)	276.19 ± 11.79 ^b^	322.22 ± 13.81 ^a^	0.030
ADG (g/d)	397.42 ± 3.48 ^b^	440.89 ± 17.45 ^a^	0.035
G/F	0.69 ± 0.03	0.73 ± 0.03	0.352
Diarrhea ratio	2.381 ± 1.37	1.984 ± 0.79	0.815

Abbreviations: ADG = average daily gain; ADF = average daily food intake; G/F = ADG/ADFI. ^a,b^ Means within a row with different superscripts differ (*p* < 0.05), the same as in the following table.

**Table 4:** Effects of LR1 on intestinal morphology of weaned piglets.

Item	CON	LR1	*p*-Value
Duodenum			
Villus height (μm)	589.33 ± 38.88	474.12 ± 39.26	0.064
Crypt depth (μm)	355.58 ± 46.37	477.69 ± 57.71	0.130
Villus height–crypt depth	1.83 ± 0.30 ^a^	1.05 ± 0.13 ^b^	0.036
Jejunum			
Villus height (μm)	457.23 ± 52.61	427.13 ± 42.41	0.665
Crypt depth (μm)	320.66 ± 22.87	394.25 ± 35.81	0.114
Villus height–crypt depth	1.47 ± 0.19	1.13 ± 0.14	0.178
Ileum			
Villus height (μm)	351.10 ± 21.23 ^b^	448.00 ± 25.79 ^a^	0.016
Crypt depth (μm)	301.43 ± 29.39	359.17 ± 19.50	0.133
Villus height–crypt depth	1.20 ± 0.09	1.27 ± 0.10	0.622

^a,b^ Means within a row with different superscripts differ (*p* < 0.05).

**Table 5:** Effects of LR1 on free amino acid concentrations in the hepatic portal vein (nmol/μL).

Amino Acids	CON	LR1	*p*-Value
Alanine	0.407 ± 0.05 ^a^	0.196 ± 0.01 ^b^	0.009
Arginine	0.029 ± 0.01	0.020 ± 0.00	0.223
Aspartic Acid	0.015 ± 0.00	0.012 ± 0.00	0.381
Asparagine	0.340 ± 0.01	0.030 ± 0.00	0.593
β-Alanine	0.007 ± 0.00	0.008 ± 0.00	0.327
Cysteine	0.007 ± 0.00 ^a^	0.003 ± 0.00 ^b^	0.020
Glutamic Acid	0.214 ± 0.04	0.123 ± 0.01	0.054
Glutamine	0.639 ± 0.07 ^a^	0.411 ± 0.03 ^b^	0.022
Glycine	0.244 ± 0.03 ^b^	0.345 ± 0.02 ^a^	0.009
Histidine	0.015 ± 0.00 ^a^	0.009 ± 0.00 ^b^	0.023
Hydroxyproline	0.019 ± 0.00 ^b^	0.027 ± 0.00 ^a^	0.031
Isoleucine	0.032 ± 0.00 ^a^	0.023 ± 0.00 ^b^	0.008
Lysine	0.050 ± 0.01	0.040 ± 0.00	0.329
Methionine	0.009 ± 0.00	0.009 ± 0.00	0.908
Ammonia	0.110 ± 0.04	0.054 ± 0.01	0.174
Ornithine	0.021 ± 0.00	0.028 ± 0.00	0.139
Phenylalanine	0.020 ± 0.00	0.020 ± 0.00	0.922
Proline	0.066 ± 0.01	0.058 ± 0.00	0.220
Serine	0.040 ± 0.00	0.043 ± 0.00	0.486
Taurine	0.074 ± 0.01	0.075 ± 0.01	0.979
Threonine	0.020 ± 0.00	0.031 ± 0.00	0.055
Tryptophan	0.021 ± 0.00	0.033 ± 0.01	0.364
Tyrosine	0.011 ± 0.00	0.013 ± 0.00	0.497
Urea	0.542 ± 0.19	0.853 ± 0.12	0.200
Valine	0.052 ± 0.01	0.046 ± 0.01	0.528

^a,b^ Means within a row with different superscripts differ (*p* < 0.05).

**Table 6:** Effects of LR1 on free amino acid concentrations in the liver (nmol/μL).

Amino Acids	CON	LR1	*p*-Value
Alanine	4.605 ± 0.11 ^a^	2.745 ± 0.16 ^b^	<0.001
Anthranilic Acid	0.024 ± 0.01	0.038 ± 0.01	0.315
Arginine	0.047 ± 0.01	0.062 ± 0.01	0.404
Aspartic Acid	1.516 ± 0.12 ^a^	1.041 ± 0.04 ^b^	0.01
Asparagine	4.544 ± 0.33	3.690 ± 0.22	0.057
β-Alanine	0.187 ± 0.02	0.180 ± 0.01	0.699
Carnitine	0.009 ± 0.00	0.009 ± 0.00	0.938
Cysteine	0.273 ± 0.04 ^a^	0.032 ± 0.01 ^b^	0.002
Cystathionine	0.016 ± 0.00	0.013 ± 0.00	0.301
Ethanolamine	0.298 ± 0.04	0.385 ± 0.06	0.263
Glutamic Acid	3.343 ± 0.22 ^a^	2.703 ± 0.07 ^b^	0.019
Glutamine	8.371 ± 0.34 ^a^	5.447 ± 0.16 ^b^	<0.001
Glycine	3.624 ± 0.28	3.290 ± 0.16	0.329
Histidine	0.484 ± 0.04 ^a^	0.301 ± 0.02 ^b^	0.002
Isoleucine	0.512 ± 0.05 ^a^	0.341 ± 0.03 ^b^	0.022
Leucine	1.135 ± 0.11 ^a^	0.776 ± 0.06 ^b^	0.015
Lysine	1.418 ± 0.12 ^a^	1.003 ± 0.09 ^b^	0.019
Methionine	0.363 ± 0.03	0.283 ± 0.02	0.071
Ammonia	4.081 ± 0.37 ^a^	3.084 ± 0.21 ^b^	0.041
Ornithine	0.892 ± 0.09 ^a^	0.612 ± 0.07 ^b^	0.029
Phenylalanine	0.425 ± 0.04 ^a^	0.258 ± 0.02 ^b^	0.003
Proline	1.035 ± 0.10 ^a^	0.760 ± 0.07 ^b^	0.045
Serine	2.053 ± 0.13 ^a^	1.697 ± 0.06 ^b^	0.032
Taurine	0.984 ± 0.06 ^b^	1.224 ± 0.04 ^a^	0.007
Threonine	0.905 ± 0.09 ^a^	0.683 ± 0.05 ^b^	0.044
Tryptophan	0.209 ± 0.04	0.171 ± 0.02	0.414
Tyrosine	0.472 ± 0.04	0.416 ± 0.04	0.329
Urea	1.569 ± 0.18	1.605 ± 0.09	0.862
Valine	1.021 ± 0.10 ^a^	0.700 ± 0.06 ^b^	0.019

^a,b^ Means within a row with different superscripts differ (*p* < 0.05).

## Data Availability

None of the data were deposited in an official repository. The data are available from the authors upon request.
